# Supplementary material for: Regulation of alternative polyadenylation by Nkx2-5 and Xrn2 during mouse heart development
Source: eLife. 2016 Jun 22;5:e16030. doi: 10.7554/eLife.16030 (PMC4982761; doi:10.7554/eLife.16030)
Supplement: Supplementary file 1. — (A) Mapping results of ChIP-seq . (B) Mapping results of RNA-seq. (C) Primers used in this study. (D) Antibodies used in this study. DOI: http://dx.doi.org/10.7554/eLife.16030.030 [file elife-16030-supp1.docx]

| **Supplementary file1A.** Mapping results of ChIP-seq | | | | |
| --- | --- | --- | --- | --- |
|  | total tag | mapped tag | %(mapped/total) | unique (4mismatches) |
| Brg1_exp1 | 9,744,740 | 4,976,539 | 51.07% | 4,412,471 (88.67%) |
| Brg1_exp2 | 30,854,944 | 12,397,601 | 40.18% | 11,090,817 (89.46%) |
| Brg1_exp3 | 46,169,109 | 26,654,994 | 57.73% | 23,341,511 (87.57%) |
| Gata4_exp1 | 78,888,135 | 42,017,560 | 53.26% | 35,277,442 ( 83.96%) |
| Gata4_exp2 | 45,425,909 | 22,566,159 | 49.68% | 19,124,221 (84.75%) |
| H3_exp1 | 39,392,724 | 17,457,716 | 44.32% | 14,678,005 (84.08%) |
| H3_exp2 | 4,751,850 | 699,777 | 14.73% | 588,972 (84.17%) |
| H3_exp3 | 45,800,472 | 28,419,609 | 62.05% | 23,810,546 (83.78%) |
| H3K27ac_exp1 | 66,335,150 | 37,573,635 | 56.64% | 33,505,510 (89.17%) |
| H3K27ac_exp2 | 44,989,997 | 31,466,399 | 69.94% | 28,983,867 (92.11%) |
| H3K27me1_exp1 | 89,314,855 | 53,043,599 | 59.39% | 46,020,138 (86.76%) |
| H3K27me1_exp2 | 52,506,234 | 34,040,334 | 64.83% | 29,491,949 (86.64%) |
| H3K27me3_exp1 | 76,053,103 | 44,905,166 | 59.04% | 39,595,531 (88.18%) |
| H3K27me3_exp2 | 30,348,324 | 19,369,270 | 63.82% | 16,893,983 (87.22%) |
| H3K36me1_exp1 | 138,605,491 | 72,348,264 | 52.20% | 63,700,525 (88.05%) |
| H3K36me1_exp2 | 61,919,941 | 37,650,074 | 60.80% | 33,341,173 (88.56%) |
| H3K36me3_exp1 | 150,268,542 | 79,278,471 | 52.76% | 62,222,182 (78.49%) |
| H3K36me3_exp2 | 36,510,536 | 22,185,482 | 60.76% | 17,792,242 (80.20%) |
| H3K4me1_exp1 | 93,176,272 | 55,657,976 | 59.73% | 51,879,409 (93.21%) |
| H3K4me1_exp2 | 43,534,462 | 28,792,389 | 66.14% | 26,886,652 (93.38%) |
| H3K4me3_exp1 | 65,238,732 | 34,086,035 | 52.25% | 29,611,939 (86.87%) |
| H3K4me3_exp2 | 34,937,547 | 22,112,977 | 63.29% | 18,183,577 (82.23%) |
| H3K9me1_exp1 | 45,036,432 | 26,281,322 | 58.36% | 22,552,905 (85.81%) |
| H3K9me1_exp2 | 42,900,473 | 28,635,697 | 66.75% | 26,209,107 (91.53%) |
| H3K9me3_exp1 | 76,947,698 | 39,223,630 | 50.97% | 20,784,918 (52.99%) |
| H3K9me3_exp2 | 55,786,406 | 32,938,984 | 59.04% | 20,785,133 (63.10%) |
| H3S28P_exp1 | 55,135,859 | 30,084,239 | 54.56% | 23,918,548 (79.51%) |
| H3S28P_exp2 | 45,216,266 | 27,216,968 | 60.19% | 20,681,755 (75.99%) |
| HDAC1_exp1 | 60,494,844 | 33,916,935 | 56.07% | 28,798,853 (84.91%) |
| HDAC1_exp2 | 55,894,801 | 31,240,848 | 55.89% | 26,707,544 (85.49%) |
| HDAC2_exp1 | 47,991,685 | 27,966,388 | 58.27% | 24,002,152 (85.82%) |
| HDAC2_exp2 | 58,254,634 | 33,088,429 | 56.80% | 28,587,837 (86.40%) |
| Input | 79,514,033 | 45,089,388 | 56.71% | 37,906,025 (84.07%) |
| Matr3_exp1 | 23,610,617 | 11,972,149 | 50.71% | 9,888,865 (82.60%) |
| Matr3_exp2 | 43,116,668 | 24,894,183 | 57.74% | 20,765,375 (83.41%) |
| Nkx2-5_exp1 | 75,577,047 | 48,431,843 | 64.08% | 44,087,073 ( 91.03%) |
| Nkx2-5_exp2 | 26,853,773 | 14,671,412 | 54.63% | 13,268,041 (90.43%) |
| Nkx2-5_exp3 | 55,366,115 | 23,659,966 | 42.73% | 21,551,515 (91.09%) |
| OGT_exp1 | 48,408,121 | 27,352,823 | 56.50% | 23,331,751 (85.30%) |
| OGT_exp2 | 9,027,388 | 4,246,851 | 47.04% | 3,503,074 (82.49%) |
| p300_exp1 | 3,034,585 | 1,020,351 | 33.62% | 807,762 (79.17%) |
| p300_exp2 | 50,028,778 | 2,978,679 | 5.95% | 2,478,715 (83.22%) |
| p300_exp3 | 11,180,761 | 6,001,480 | 53.68% | 5,011,966 (83.51%) |
| p300_exp4 | 33,522,230 | 18,920,167 | 56.44% | 15,748,580 (83.24%) |
| Raver1_exp1 | 17,612,235 | 9,748,339 | 55.35% | 8,210,857 (84.23%) |
| Raver1_exp2 | 32,154,914 | 17,118,801 | 53.24% | 14,915,557 (87.13%) |
| RNAPII-S2P_exp1 | 58,400,005 | 29,805,473 | 51.04% | 26,969,867 (90.49%) |
| RNAPII-S2P_exp2 | 63,164,532 | 37,655,143 | 59.61% | 34,179,331 (90.77%) |
| RNAPII-S5P_exp1 | 36,501,916 | 17,422,968 | 47.73% | 15,938,974 (91.48%) |
| RNAPII-S5P_exp2 | 12,618,622 | 2,313,774 | 18.34% | 2,125,046 (91.84%) |
| RNAPII-S5P_exp3 | 30,855,779 | 17,333,671 | 56.18% | 15,580,409 (89.89%) |
| Tbx5_exp1 | 80,358,417 | 49,794,187 | 61.97% | 45,103,772 ( 90.58%) |
| Tbx5_exp2 | 25,498,855 | 13,364,731 | 52.41% | 11,963,198 (89.51%) |
| Tbx5_exp3 | 47,368,448 | 19,515,381 | 41.20% | 17,626,854 (90.32%) |
| Whsc1_exp1 | 48,219,842 | 24,503,321 | 50.82% | 18,305,226 (74.71%) |
| Whsc1_exp2 | 61,908,460 | 34,723,194 | 56.09% | 28,598,331 (82.36%) |

| **Supplementary file1B.** Mapping results of RNA-seq | | |
| --- | --- | --- |
|  | Total reads | Reads mapped |
| WT_exp1 | 67,092,292 | 51,479,733 |
| siControl_exp1 | 62,632,307 | 49,281,509 |
| siNkx2-5_exp1 | 85,829,056 | 58,809,730 |
| siTbx5_exp1 | 68,314,913 | 53,119,403 |
| siGata4_exp1 | 82,542,247 | 57,976,180 |
| siXrn2_exp1 | 104,943,121 | 78,547,122 |
| WT_exp2 | 88,222,504 | 64,361,780 |
| siControl_exp2 | 93,334,440 | 66,494,854 |
| siNkx2-5_exp2 | 84,943,191 | 61,448,569 |
| siTbx5_exp2 | 78,221,449 | 54,799,822 |
| siGata4_exp2 | 74,766,183 | 58,026,756 |
| siXrn2_exp2 | 115,306,498 | 89,651,394 |
| Nkx_WT (E9.5)__exp1 | 65,656,237 | 52,618,079 |
| Nkx_WT (E9.5)__exp2 | 77,491,456 | 63,530,205 |
| Nkx_KO (E9.5)__exp1 | 56,523,425 | 45,543,796 |
| Nkx_KO (E9.5)__exp2 | 56,751,758 | 46,082,586 |
| siControl_chrRNA | 17,452,512 | 13,111,189 |
| siNkx2-5_chrRNA | 24,270,935 | 17,492,417 |
| siXrn2_chrRNA | 18,981,848 | 14,046,210 |

**Supplementary file1C.** Primers used in this study

| ATP2a2-ChIP5.2-S  ATP2a2-ChIP5.2-AS | TCGGGGTATCAAAGTGGAGA  ACGTGTCAGGAGGAAAGCAG | ChIP-qPCR for ATP2a2-TSS | |
| --- | --- | --- | --- |
| ATP2a2-3-S  ATP2a2-3-AS | TTGTGACTTGATGGTTCTCACC  AGGTTGTAAGCAAAGGACTCCA | qRT-PCR, ChIP-qPCR, and 3C for ATP2a2-3 | |
| Atp2a2-3UTRtoDown-S  Atp2a2-3UTRtoDown-AS | CACAGCGCATGTTTGACTGT  CTGTGACACGCATTCTGCTT | ChIP-qPCR for 3’UTR of Atp2a2 | |
| Tnnt2-3-S  Tnnt2-3-AS | CCGAGTCACCTTGTAGCTCTCT  ACAACCTAGAGAGATGGGCAGA | qRT-PCR, ChIP-qPCR, and 3C for Tnnt2-3 | |
| Tnnt2-exon1-S  Tnnt2-exon1-AS | CACATGCCTGCTTAAAGCTCTC  TGCACACAGGTCTTGAGGTATC | ChIP-qPCR for Tnnt2-TSS | |
| Tnnt2-3UTRtoDown-S  Tnnt2-3UTRtoDown-AS | AAAAGCCAACACACTGCACA  AAACATAAACCCTGCCCTCTG | ChIP-qPCR for 3’UTR of Tnnt2 | |
| Tnnt2-exon10-S  Tnnt2-exon10-AS | AGGAGGAGGAGAACAGGAGGAA  TCTGGATGTACCCTCCAAAGTG | ChIP-qPCR for the exon 10 of Tnnt2 | |
| Myl7-TSS-S  Myl7-TSS-AS | GCTTCCAAGTTTAGCCCAATC  CCACCCAGCTCTTTATGTCTCA | ChIP-qPCR for the TSS of Myl7 | |
| Myl7-exon4-S  Myl7-exon4-AS | CATCAACTTCACCGTCTTCCTC TGGCTAAGGAGACTCAGACCTC | ChIP-qPCR for the exon 4 of Myl7 | |
| Myl7-TTS-S  Myl7-TTS-AS | CTGCAGGTAGAGCAACTGTTTG  GTGGGTGATGATGTAGCAGAGA | ChIP-qPCR for the TTS of Myl7 | |
| Myl7-3-S  Myl7-AS | AATGCTGAAAACTGGTCTCCTG  AGGGAGATATGGGGAGACTAACA | ChIP-qPCR for the downstream of Myl7 | |
| Rplp2-i2-S  Rplp2-i2-AS | CTTGTGCAATCGAAGAAGTCAG  TCCAGGTTAGCCAGAGCTACA | qRT-PCR for Rplp2 in chromatin RNA | |
| ATP2a2-RT3-S  ATP2a2-RT3-AS | tggtgatatagtggaaattgctg  gagttgtagacttgatggatgtcaa | qRT-PCR for Atp2a2 | |
| Tnnt2-S  Tnnt2-AS | TTCGACCTGCAGGAAAAGTT  CTTCCCACGAGTTTTGGAGA | qRT-PCR for Tnnt2 | |
| H1foo-promoter-S  H1foo-promoter-AS | AGGCTAGCAGTAGTCTGGATCAG  ACTGTGTCCTACCTACCTGACGAG | ChIP-qPCR for H1foo | |
| Tnnt2-frag1-3C | CTTGCATTCTTGACGGGAAGTGAGTTGG | 3C for Tnnt2, anchoring | |
| Tnnt2-frag3-3C | CGAAACTTGCAACTCTCATGCTGGAAGG | 3C for Tnnt2, 4.3 kb | |
| Tnnt2-frag5-3C | CCTATAAACACAGCCAGCCATTCTCTGC | 3C for Tnnt2, 9.8 kb | |
| Tnnt2-frag6-3C | CCGGGATCCAGAAGCTAATGATGTGTCATGC | 3C for Tnnt2, 14.0 kb | |
| Tnnt2-frag9-3C_2 | GGAAGCCCTTCTAAGTTCAATAGACAAGGG | 3C for Tnnt2, 18.4 kb | |
| Atp2a2-frag2-3C | GGGGGGAAAATGATAGAAGCTGTTTTGC | 3C for Atp2a2, anchoring | |
| Atp2a2-frag9-3C | GTATCGACAGGACAGAAAGAGTGTGCAACG | 3C for Atp2a2, 11.3 kb | |
| Atp2a2-frag12-3C | GATAGACACTGATTGGAGCCTGGTTAGG | 3C for Atp2a2, 32.3 kb | |
| Atp2a2-3C_frag14-3 | GTGGTTAGCATTGCTTGTTACCAGGACACAGC | 3C for Atp2a2, 36.4 kb | |
| Atp2a2-3C_frag16_4 | GGCCTTTTCAGCTCAGACTAGAAGTCTACACG | 3C for Atp2a2, 47.2 kb | |
| Tnni1-3C-4554-S | CAAGGCATGGACTCTAAGTGCT | 3C for Tnni1, anchoring | |
| Tnni1-3C-4554-AS | ACCCAACACACTAGGCTACGAC | 3C for Tnni1, 2.7 kb | |
| Tnni1-3C-6943-AS | GGGGAGAGGAAGGGGTATAGTT | 3C for Tnni1, 5.1 kb | |
| Tnni1-3C-13751-AS | GAACTTGGCTTTTTAGGGTGGT | 3C for Tnni1, 11.9 kb | |
| Nkx2-5-RT-S  Nkx-2-5-RT-AS | GACGTAGCCTGGTGTCTCG  GTGTGGAATCCGTCGAAAGT | qRT-PCR for Nkx2-5 | |
| GATA4-RT-S  GATA4-RT-AS | GGAAGACACCCCAATCTCG  CATGGCCCCACAATTGAC | qRT-PCR for Gata4 | |
| RPLP2-qPCR-S  RPLP2-qPCR-AS | TCTCCTAGCGCCAAAGACAT  CCATTCAGCTCACTGATGACC | | qRT-PCR for Rplp2 |
| Xrn2-RT1-S  Xrn2-RT1-AS | GCAGAAGACAGTGACAGTGAGC  CAACCAGCTTCCCATAACCT | | qRT-PCR for Xrn2 |
| Xrn2-gw-S  Xrn2-gw-AS | CACCATGGGAGTCCCGGCGTTCTT  CTAATTCCAACTGTATCTTCCCGAGGGT | | Cloning for Xrn2 |
| Xrn2-XRNdel-5 | CACCATGAAACCAAATAAACCTAAACCAT | | Cloning for Xrn2ΔXRN |
| Xrn2-Cdel-3 | CTAACCTTCACAATCCTTGACCTCGTGT | | Cloning for Xrn2ΔC |
| Xrn2-HindZindel-5  Xrn2-HindZindel-3 | ACCAAGCTTTTGCCAAGAGAAAAGAAGGGA  CCCAAGCTTTTTAGGTTTATTTGGTTTGA | | Cloning for Xrn2ΔZF |
| Tnnt2-probe-S  Tnnt2-probe-AS | AAGAAGGTCCAGTAGAGGACACC  GCCAGGATCTTCTTCTTCTTCTC | | Probe for Northern blot |
| Atp2a2-probe4-S  Atp2a2-probe4-AS | TACAGCCTTTGTAGAGCCGTTT  TGTAGGGGTGTTCTCTCCTGTT | | Probe for Northern blot |
| Nodal-qPCR-Fw  Nodal-qPCR-Rv | CCAACCATGCCTACATCCA  CACAGCACGTGGAAGGAAC | | qRT-PCR for Nodal |
| Lefty-qPCR-Fw  Lefty-qPCR-Rv | ACTCAGTATGTGGCCCTGCTA  AACCTGCCTGCCACCTCT | | qRT-PCR for Lefty |
| Pitx2-qPCR-Fw  Pitx2-qPCR-Rv | CCTTACGGAAGCCCGAGT  AAAGCCATTCTTGCACAGC | | qRT-PCR for Pitx2 |
| Cryptic-qPCR-Fw  Cryptic-qPCR-Rv | GCCTATGGGATTCCCTTCC  ACAGCGGGATACAGGGACT | | qRT-PCR for Cryptic |

**Supplementary fileD.** Antibodies used in this study.

| Antibodies (anti-) | Source (Cat#) | Source (company) | Application |
| --- | --- | --- | --- |
| Nkx2-5 | N-19 | Santa Cruz Biotechnology | ChIP-seq, IP, WB |
| Tbx5 | ab18531 | Abcam | ChIP-seq, IP, WB |
|  | AF5918 | R&D systems | WB |
| Gata4 | G-4 | Santa Cruz Biotechnology | ChIP-seq, IP, WB |
| RNAPII | N-20 | Santa Cruz Biotechnology | IP, WB |
| RNAPII-S2P | ab5095 | Abcam | ChIP-seq |
|  | H5 | COVANCE | WB |
| RNAPII-S5P | ab5131 | Abcam | ChIP-seq |
|  | H14 | COVANCE | WB |
| p300 | C-20 | Santa Cruz Biotechnology | ChIP-seq, WB |
| HYPB | ab31358 | Abcam | WB |
| Whsc1 | Kaneda lab. |  | ChIP-seq, WB |
| NSD3 | ab4514 | Abcam | WB |
| Brg1 | H-88 | Santa Cruz Biotechnology | ChIP-seq, WB |
| HDAC1 | ab7028 | Abcam | ChIP-seq, WB |
| HDAC2 | ab7029 | Abcam | ChIP-seq, WB |
| OGT | H-300 | Santa Cruz Biotechnology | ChIP-seq, WB |
| H3 | ab1791 | Abcam | ChIP-seq, WB |
| H3K4me1 | ab8895 | Abcam | ChIP-seq |
| H3K4me3 | ab8580 | Abcam | ChIP-seq |
| H3K9me1 | ab9045 | Abcam | ChIP-seq |
| H3K9me3 | ab8898 | Abcam | ChIP-seq |
| H3K27me1 | 07-448 | MILLIPORE | ChIP-seq |
| H3K27me3 | 07-449 | MILLIPORE | ChIP-seq |
| H3K36me1 | ab9048 | Abcam | ChIP-seq |
| H3K36me3 | ab9050 | Abcam | ChIP-seq |
| H3K27ac | ab4729 | Abcam | ChIP-seq |
| H3S28P | 07-145 | MILLIPORE | ChIP-seq |
| Matrin3 | Kaneda lab. |  | ChIP-seq, WB |
| Raver1 | Kaneda lab. |  | ChIP-seq, WB |
| Xrn2 | A301-103A  A301-101A | BETHYL laboratoryies  　BETHYL laboratoryies | IP, ChIP  WB |
| Senataxin | ab56984 | Abcam | WB |
| DHX9 | ab26271 | Abcam | WB |
| DDX5 | ab21696 | Abcam | WB |
| M2-HRP | A8592 | SIGMA-ALDRICH | WB |
| M2 | A2220 | SIGMA-ALDRICH | IP |
| HA | H9658 | SIGMA-ALDRICH | WB |
| HA | E6779 | SIGMA-ALDRICH | IP |
